# A Numerical Study of Crack Mixed Mode Model in Concrete Material Subjected to Cyclic Loading

**DOI:** 10.3390/ma16051916

**Published:** 2023-02-25

**Authors:** Omar Alrayes, Carsten Könke, Khader M. Hamdia

**Affiliations:** 1 Institute of Structural Mechanics, Bauhaus Weimar University, Marienstraße 15, 99423 Weimar, Germany; 2 Institute of Continuum Mechanics, Leibniz Universität Hannover, 30167 Hannover, Germany

**Keywords:** mixed mode crack propagation, cohesive zone method, cyclic loading, SBFEM

## Abstract

In quasi-brittle materials such as concrete, numerical methods are frequently used to simulate the crack propagation for monotonic loading. However, further research and action are required to better understand the fracture properties under cyclic loading. For this purpose, in this study, we present numerical simulations of mixed-mode crack propagation in concrete using the scaled boundary finite element method (SBFEM). The crack propagation is developed based on a cohesive crack approach combined with the thermodynamic framework of a constitutive concrete model. For validation, two benchmark crack-mode examples are modelled under monotonic and cyclic loading conditions. The numerical results are compared against the results from available publications. Our approach revealed good consistency compared to the test measurements from the literature. The damage accumulation parameter was the most influential variable on the load-displacement results. The proposed method can provide a further investigation of crack growth propagation and damage accumulation for cyclic loading within the SBFEM framework.

## 1. Introduction

The application of fatigue fractures is essential in analysing the performance of concrete structures. In fracture mechanics, concrete discontinuities also have the most significant investigation in the field of engineering [1,2]. To better understand the rapid failure of concrete structures under cyclic loading, a detailed procedure of fatigue crack propagation is required. The prediction of the direction of crack propagation and orientation of quasi-brittle material as concrete is essential for the robust and reliable design of concrete structures.

In concrete material, modelling of crack propagation and the numerical simulation of crack growth remains an outstanding issue and a critical topic of ongoing research. Primarily, the finite element technique is mainly used to simulate the crack behaviour numerically. Still, discontinuities in material simulation cannot be fully demonstrated, since the finite element method (FEM) is based on a continuum approach.

The cracks are typically mapped by areas of high strain rates when using the smeared crack approach, as in Ref. [3]. The division of the crack opening into an equivalent element length of a finite element causes the effect of smeared crack formation. This method has a drawback in that it cannot accurately reflect the actual fracture pattern because the distortion and discontinuity in the displacement field are not mapped. Alternately, discontinuities are added at the element edges in the discrete crack approach [4]. This method is affiliated with a high numerical effort since each iteration step has a continuous re-meshing process.

Based on the extensions of the conventional FEM, cohesive numerical approaches in modelling crack propagation have been developed to avoid this disadvantage [5,6,7,8]. Particular crack tip components were created to reduce the mesh quality essential for crack simulation by the FEM [9]. However, many difficulties have been reported by Ref. [10] for material modelling using the FEM framework. The nodal displacements for finite elements at the crack tip should be omitted when calculating the stress intensity factor (SIFs). To determine the crack propagation path, several theories have been put forward [11]. Since it has an approximate explicit solution for the crack growth direction (θ) as a function of the stress intensity factor under pure tension ($K_{I}$) and mixed-mode condition ($K_{II}$), the maximum tangential stress criterion is frequently used in FEM simulations of cyclic crack propagation. In this case, it should be noted that the procedure of mesh refinement is typically needed in the vicinity of the crack tip. The global or local re-meshing technique is the most standard method to describe monotonic and cyclic crack propagation under linear elastic fracture mechanics (LEFM) assumptions [11].

The cohesive zone model of Ref. [12] is most commonly used to model the process zone. The process zone is often modelled in FEM utilizing the zero-thickness interface elements. Interface elements are used in a variety of modelling techniques in the literature, such as placing them along the crack paths [13,14], inserting them along all element interfaces in the mesh [15,16,17], and placing them along the crack surfaces as the crack propagates [18,19,20]. While some methods are derived based on a priori information of the crack paths obtained from experiments [13,14,21], sophisticated re-meshing algorithms to propagate the crack with high mesh densities or particular finite elements were implemented to model the singular stress fields around crack tips [22,23]. High mesh densities are needed to achieve smooth and precise predicted crack paths, even though the methods developed in Refs. [15,16,17,24] do not. An additional nodal enrichment with special stress functions was included by using the extended finite element method (XFEM) to simulate singular stress zones around crack tip [25]. The same appealing property that does not require re-mesh to describe crack propagation is shared by XFEM and embedded crack models. The cohesive tractions at the crack edges are included in the governing equations to account for their work. Many research investigations have discussed cohesive crack propagation for statics and dynamics issues using XFEM [26,27].

Meanwhile, the scaled boundary finite element method (SBFEM) has been proposed recently to facilitate the dilemma of the computation burdens [28,29,30,31]. The SBFEM is a very efficient method in solving problems with unbounded media and singularities. The method’s effectiveness in handling singularities and unbounded domain problems has prompted researchers to extend its applications to solve diverse problems in various engineering fields, such as fracture mechanics [32,33], dam reservoir interaction [34], electromagnetic [35] and image-based analysis [36]. In order to minimize computation costs, the SBFEM assigns no discretization of side-face boundaries [33]. Using polygon elements created by the SBFEM, Ref. [37] has developed an automatic LEFM-based crack propagation modelling technique. By utilizing SBFEM’s appealing feature, the particular stress fields near crack tips were analytically represented [28]. The SBFEM has shown a considerable efficiency compared to the classical FEM in calculating the stress singularities [38]. Crack-tip mesh refinement, as in FEM, is avoided since the SBFEM calculates SIFs from the stress solutions at the edges of subdomains and the nodes on the domain boundary. Furthermore, a domain can be divided into subdomains in any required way, and the accuracy of the stress and SIFs solutions is specified based on the re-meshing procedure. Accordingly, this feature is more flexible in simulation crack propagation than in the FEM. In addition, the re-meshing procedure can be as simple as used in the boundary element method (BEM). Egger et al. [39] examined the computational efficiency of the SBFEM for solving linear elastic fracture mechanics problems. A comparison between the SBFEM, Extended finite element method (XFEM), and FEM was constructed by introducing different examples for calculating the SIFs. The output showed that the SBFEM reached the exact solution faster than XFEM. An extended finite element method by Ref. [40] was developed to simulate nonlinear dynamic analysis. A direct remeshing algorithm for crack propagation has been obtained in quasi-brittle materials. However, more investigations are required in modelling crack propagation for concrete under cyclic and fatigue loading. As the singularities of cracks in the material interface are analytically calculated, the initial crack and the stress state can be easily defined.

In this study, the SBFEM framework developed in Ref. [32] is implemented to model cohesive crack propagation in quasi-brittle materials under cyclic loading. A new constitutive model based on Ref. [41] is implemented to model the propagating cracks that depend on the direction of applied loads. This model introduces an efficient simulation of concrete material under cyclic behaviour. At sub-critical loading levels, the model relies on the cumulative measure of propagation as a key damage-driving mechanism. The results of the proposed approach are validated against the methods in Refs. [42,43]. Concerning the concrete elements’ fracture and material response, analyses are performed using the thermodynamic constitutive material law for concrete. The material model assumes a combination of plasticity and damage theory in Refs. [44,45,46]. Similarly, as in Ref. [47] the proposed method aims to simulate the crack propagation of concrete under cyclic loading. However, we further extend the SBFEM framework to simulate the mixed mode crack damage behaviour under various loading scenarios. As results, two mixed-mode crack propagation problems are modelled for monotonic and cyclic loading. The results are discussed and compared with the data available in publications.

The paper is structured as follows. In Section 2, the principle of the constitutive material model at the cohesive interface element is explained. In addition, the constitutive material model at the cohesive interface element for mixed-mode material response is investigated. Section 3 introduces the proposed mixed-mode crack procedure for concrete material using SBFEM. Additionally, the re-meshing procedure of crack propagation is introduced. Section 4 introduces a nonlinear crack model for cohesive interactions, and a flowchart for solving the SIFs is given. In Section 5, two numerical simulations are modelled to validate the nonlinear model. Based on the findings of the cyclic bending test of plain concrete that were published in the literature, we provide the calibration and validation of the proposed model. The effect of the loading sequence on the material’s stiffness was the main focus of the numerical investigations.

## 2. Constitutive Relation under Cyclic Loading

The advanced material models define a direct relationship between the invariant of the strain and stress by linking the damage evolution with the strain, as in Refs. [10,48,49]. In order to reflect the opening/closure and growth of the micro-cracks and/or the frictional sliding along their length, the formulation of the dissipative mechanisms has been refined by introducing the internal sliding strain as a damage-driving variable within the framework of isotropic damage and internal sliding strain. This way, a unified model for monotonic and low cycle fatigue loads was proposed by Refs. [50,51].

**Figure 1:**
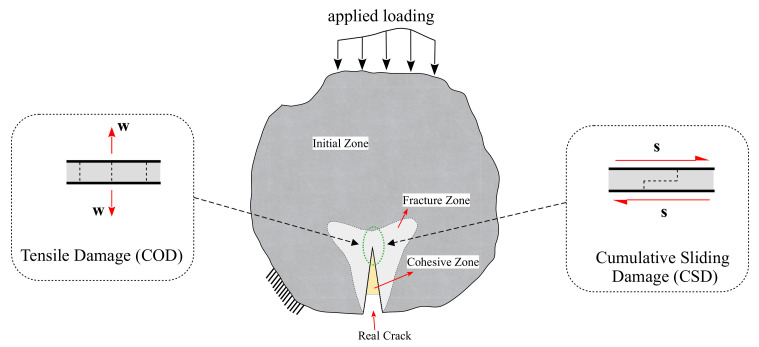
Modelling approach of the cohesive concrete zone for both crack opening and sliding.

Comparing the thermodynamic softening law of the constitutive model for the fracture, the proposed model has the ability to simulate the plastic deformation of the experimental results based on the plasticity and damage variables. Therefore, a numerical approach is introduced in this work to consider monotonic and cyclic behaviour. The proposed approach considers the cumulative measure of slip as an essential damage-driving mechanism at the subcritical loading levels, as illustrated in Figure 1. The constitutive behaviour of the embedded interface elements that represent the fracture process zone has been identified utilizing the thermodynamic-based interface model [1,47].

A cumulative measure of the inelastic displacement inside the interface governs the damaged evolution of concrete material. The evolution law in Equation (1) introduces the cumulative opening and/or sliding as the fundamental source of damage. The adopted failure criterion is identified based on the evolution law of the threshold function of the damage plasticty law, as described in Ref. [41]. In a manner similar to how Lemaitre’s damage potential was presented in Ref. [52], this feature has been introduced through the modified flow potential in Refs. [50,53].

(1) $$\dot{\omega }={\left(1-\omega \right)}^{c+1}{\left(\frac{Y}{S}\right)}^{r}\left(\frac{\sigma_{n}}{\tilde{\sigma_{n}}-m\sigma_{0}}\right)\left|{\dot{u}}^{P}\right|$$

with

(2) $${\dot{u}}^{P}=\frac{\dot{\lambda }}{1-\omega }\;\mathrm{sign}\left(\tilde{\sigma_{n}}-X\right)$$

where ${\dot{u}}^{P}$ is a representation of the relative displacement at the interface (i.e., opening displacement w=COD in the normal direction and slip s=CSD in the tangential/shear direction, see Figure 1), *Y* is the energy release rate related to the damage mechanism, and *X* represent the thermodynamic force. The state variables of the damage variable ω, *S* are the damage strength parameter, and c,r are the exponential parameters controlling the accumulation rate of the damage. The $\tilde{\sigma_{n}}$, $\sigma_{0}$ and $\sigma_{n}$ are the effective stress limits.

The introduced material model has been integrated as an implicit time-stepping method into the Scaled Boundary Finite Element Framework. The return mapping procedure is used to correct the internal variables after an elastic trial step, and the incremental multiplier at each time step is computed numerically from the consistency condition, $\dot{f}$. The exact process has been applied to the finite element framework by Ref. [41].

The incremental value Δλ can be obtained by substituting the evolution equations into the consistency condition as in Refs. [47,51].

(3) $$\Delta \lambda =\frac{f_{n+1}^{\mathrm{trial}}}{E/(1-\omega_{n})+\gamma +K}$$

where *E* is the elastic stiffness, *K* and γ represent the isotropic and kinematic hardening moduli, respectively. Due to the implicit form of the damage evolution Equation (1), the iterative Newton scheme is applied to identify an admissible state. For fatigue and cyclic simulations, this might be too expensive. As a result, we adopt the assumption of a damage quasi-constant over a time step in Refs. [41,54], which significantly speeds up the simulation without sacrificing accuracy. The proposed model needs a consistent algorithmic stiffness to ensure a reliable and effective numerical implementation. The algorithmic stiffness establishes a relationship between the rates of stress and displacement by

(4) $$\dot{\sigma }=\left(E^{alg}\right)\dot{u}$$

The stress rate then can be expressed as

(5) $$\sigma =\left(1-\omega \right)E\left(\dot{u}-{\dot{u}}^{P}\right)-\dot{\omega }E\left(u-u^{P}\right)$$

The algorithmic stiffness is obtained by substituting the evolution equations for damage and displacement with the incremental multiplier in Equation (3) as

(6) $$\begin{array}{cc} E^{alg}= & \left(1-\omega \right)E-\frac{\left(1-\omega \right)E^{2}}{E+(\gamma +K)(1-\omega )}\\ \; & -\frac{{\left(1-\omega \right)}^{c}E^{2}\left(u-u^{p}\right)\left(\frac{\bar{\sigma }}{\bar{\sigma }-m\sigma_{0}}\right){\left(\frac{Y}{S}\right)}^{r}\mathrm{sign}\left({\tilde{\sigma }}_{n+1}^{p,\mathrm{trial}}-\gamma \alpha_{n}\right)}{\left(E/(1-\omega )+\gamma +K\right)} \end{array}$$

Following the formulation of the equilibrium condition on a zero-thickness element of the interface, the described material model is embedded into the initial boundary value problem of the SBFEM in a usual manner.

## 3. Modelling Crack Propagation

### 3.1. Crack Tip Stress Field in the Presence of Cohesive Traction

**Figure 2:**
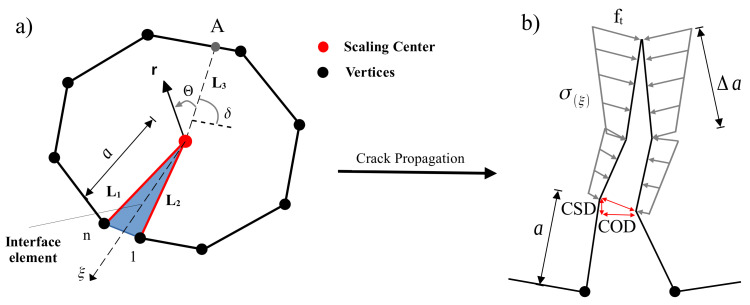
Crack propagation of interface SBFEM element: (**a**) interface cohesive model in SBFEM, (**b**) distribution of the cohesive forces.

Fracture in quasi-brittle materials such as concrete involves a process zone [55]. The numerical models and simulations consider the cracking phenomenon that can be detected physically. Due to surface friction and aggregate interlocking, normal and shear tractions can be transferred across crack surfaces. In this study, the interface elements are utilized to model the cohesive cracks that result from mixed-mode loading scenarios. Figure 2 illustrates a typical bounded domain of an interface element at the crack tip. The crack displacement in Figure 2b along the interface elements consists of crack opening displacement (COD) and crack sliding displacement (CSD). The nonlinear cohesive tractions for normal traction and tangential cohesion are σ and τ, respectively.

The governing equations of SBFEM for an element containing a crack tip with side face tractions is motivated by the works in Ref. [38]. Along the radial lines, ξ nodal displacement functions u(ξ) are used, while the displacement functions in the η direction are interpolated by the shape functions [N(η)]. The displacement field u(ξ,η) is scaled as boundary coordinates and expressed including the normal displacement modes and the sideface displacement modes as

(7) $$\left\{u(\xi ,\eta )\right\}=\left[N\left(\eta \right)\right]\sum_{i=1}^{N+M}c_{i}\xi^{\left(\bar{\lambda_{i}}-1\right)}\left\{\bar{\phi_{i}}\right\}$$

where $\bar{\phi_{i}}$ is the side-face load mode, $c_{i}$ is the integration constants, and $\bar{\lambda_{i}}$ is the eigenvalue matrix.

The stress field can be calculated in the presence of cohesive traction as

(8) $$\left\{\sigma (\xi ,\eta )\right\}=\sum_{i=1}^{N+M}c_{i}\xi^{\left(\bar{\lambda_{i}}-1\right)}\left\{\psi_{i}\left(\eta \right)\right\}$$

where each term in Equation (8) can be interpreted as a stress mode and

(9) $$\left\{\psi_{i}\left(\eta \right)\right\}=\left[D\right]\left(\bar{\lambda_{i}}\left[B_{1}\left(\eta \right)\right]+\left[B_{2}\left(\eta \right)\right]\right)\left\{\bar{\phi_{i}}\right\}$$

where [D] is the material constitutive matrix [43], $\left[B_{1}\left(\eta \right)\right]$ and $\left[B_{2}\left(\eta \right)\right]$ are the SBFEM strain-displacement matrices. In addition, it indicates that *N* is the number of displacement modes and stress modes, where an extra *M* are added to both fields when the cohesive traction is considered.

The stress intensity factors for the homogeneous material square root singular problem are defined as

(10) $$\left\{\begin{array}{c} K_{I}\\ K_{II} \end{array}\right\}=\sqrt{2\pi L_{0}}\sum_{i=I,II}\left(c_{i}{\left\{\begin{array}{c} \xi^{-\lambda_{i}-1}\sigma_{yy}{|}_{\theta =0}\\ \xi^{-\lambda_{i}-1}\sigma_{xy}{|}_{\theta =0} \end{array}\right\}}_{i}\right)$$

where $L_{0}=L_{3}$ is the distance between the crack tip and the point A at the crack surface direction on the boundary, see Figure 2a. $c_{i}$ are integration constants. As ξ→0, two modes can yield singular stresses with $\lambda_{i}$ = 0.5. These two stress modes will be considered as mode I and mode II.

### 3.2. Crack Propagation

The crack initiation in the SBFEM domain is determined according to the zero-K condition [56]. Once the stress at the crack tip is finite, a cohesive crack will propagate. Any crack that satisfies the zero-K condition at the end of each load step will be identified using the SBFEM-based algorithm created by Ref. [57]. This hypothesis assumes that a cohesive crack will propagate if there is no stress singularity and finite stress at the crack tip. The crack propagates under the following condition

(11) $$K_{I}\left(\theta \right)\geq 0$$

**Figure 3:**
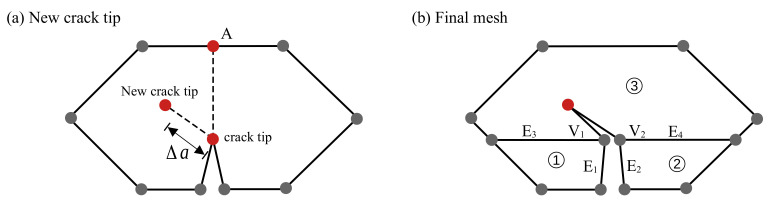
Crack propagation re-meshing procedure.

The procedure of crack propagation is illustrated in Figure 3 and is described as follows. In order to locate the new crack tip in each crack propagation step, it is necessary to first identify the crack propagation direction ($\Theta_{c}$) and the specified crack propagation length (Δa). Once the stress intensity factors (SIFs) have been calculated from Equation (10), the $\Theta_{c}$ can be computed as in Ref. [58].

The re-meshing procedure is outlined in Figure 3a,b for one crack propagation step. Given Δa and $\Theta_{c}$, the location of the new crack tip, shown in Figure 3a, is calculated and located in the cracked subdomain (point A is used to compute the SIFs). Two new vertices ($V_{1}$ and $V_{2}$ in Figure 3b) have been created from the former crack tip. Four new edges ($E_{1}$–$E_{4}$) are constructed along with the creation of two new subdomains (1 and 2). All edges of the newly cracked subdomain (3) must be visible from the new crack tip, and the new edges and subdomains are utilized to track the crack path.

The crack propagation criteria are examined when the external load increases. Once it is satisfied at a particular load, the crack length Δa and the crack angle θ are utilized to pinpoint the location of the new crack tip in the mesh as in Ref. [33].

The cohesive tractions along the crack are obtained based on the condition $K_{I}\geq 0$. The crack subdomain is split up into standard subdomains called cell interface elements (CIEs). The new CIEs are then coupled with SBFEM normal cells. The system stiffness matrix can be created by assembling the stiffness matrices and equivalent nodal forces of the subdomains and CIEs. The SIFs can then be calculated once the nodal displacements and cohesive tractions along the crack are identified. The material softening is represented by the constitutive material model, explained in Section 2.

## 4. Implementation Procedure by SBFEM

**Figure 4:**
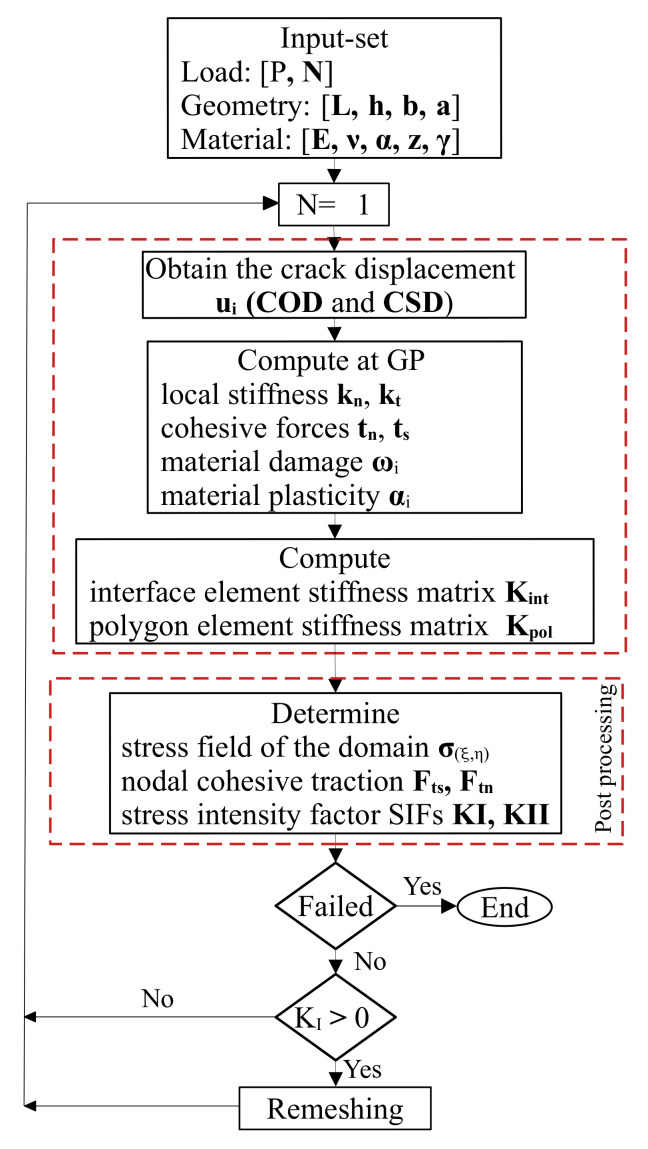
Key steps of the stress field domain and stress intensity factor SIFs.

The flow chart for the numerical process is shown in Figure 4. A further explanation of the numerical procedure is presented as follows:

1. Input the geometric dimensions of the specimen including; the span length *L*, height *h*, width *b*, and initial crack length *a*, along with the material parameters; the initial fracture toughness, Poisson’s ratio ν, Young’s modulus *E*, damage parameter ω, and the material plasticity α,γ and *z* under both static and cyclic loading *P*;
2. Establish the model (SBFEM) with the initial crack length *a*. Apply the external load *P*. Calculate the stress field of the domain, cohesive nodal traction, and the stress intensity factors (SIFs) $K_{I}$ and $K_{II}$. Adjust the applied load until the initial cracking is reached;
3. Re-establish the SBFEM of the crack angle θ with crack length *a*. Δa is the increment of crack length. If i=1, the number of cyclic loading $N_{1}=1$. Apply cyclic load $P_{max}$ and the cohesive force according to Equation (13). Finally, the single and mixed mode $K_{I}$ and $K_{II}$ for monotonic and cyclic crack propagation process can be calculated according to Equation (10);
4. Repeat step 3 until the structure fails and the numerical simulation is terminated. Output the necessary parameters, such as the crack propagation path, the number of cyclic loads *N*, and CMOD and CMSD displacements.

The above modelling methodology has been implemented in a computer program using MATLAB software. Figure 4 shows a proposed flowchart of the program. The pre-processing step is to define the input set of the tested problem. The constitutive law is inserted into the SBFEM framework as an interface element at the crack tip. The nonlinear consistent interface model is solved using the displacement control algorithm to obtain the post-processing findings for monotonic and cyclic loading. The cyclic damage accumulation during loading and unloading is formulated within the constitutive model.

Based on the constitutive model at the material point level, the relative displacement of the crack surface $u_{i}$ is calculated, including the opening displacement (COD) and the sliding displacement (CSD) of the crack surface. The key concept behind this method is the linear superposition of an iterative methodology applied to the relative displacement of the crack surface in order to solve and estimate the cohesive tractions on the crack surface.

The standard SBFEM solution of the stress intensity factor formula can calculate all three stress intensity factors.

(12) $$K_{I}=K_{I}^{P}+K_{I}^{C}$$

where $K_{I}$ is the total stress intensity factor and $K_{I}^{P}$ and $K_{I}^{C}$ are the components related to the external and cohesive forces, respectively. Thus, $K_{I}^{P}>0$ when the crack expands due to the external force of the model, while $K_{I}^{C}<0$ when the crack tends to close due to the cohesive force. When force balance is achieved as a result of the aspects covered by the external and cohesive forces, $K_{I}=0$, equivalently. Therefore, $K_{I}\geq 0$ can be utilized as the criterion for considering whether the crack will continue to propagate or not as in Ref. [59]. The solution of the cohesive tractions is summarized with the following steps:

(a) As shown in Figure 4, the linear elastic assumptions of SBFEM can be used to determine the relative displacement $u_{i}$ of the crack element when the structure is subject to the external force *P*. As a result, the corresponding cohesive traction $t_{i}$ can be acquired;
(b) Both the external force and the cohesive force obtained in the previous step are applied to the structure, with the cohesive traction $t_{i}$ being applied in the form of a side-face force and formulated in accordance with Equation (13). Along the fracture process region, cohesive tractions $t_{n},t_{s}$ are related to the relative opening and sliding displacements on the crack faces *u*
  (13) {t}=[k]{u}
  where *k* is the stiffness of the softening laws.
  The stiffness matrix of an interface element in the local coordinate system is:
  (14) $$\left[k_{int}\right]=\frac{A}{2}\sum_{i=1}^{n_{g}}w_{i}{M_{i}}^{T}\left[k\right]M_{i}$$
  where *A* is the crack surface area, $w_{i}$ is the one-dimensional Gaussian weight, $n_{g}$ is the number of integration points, and $M_{i}$ is the linear shape function matrix [33]. Based on Equation (14) the solution of the displacement and stress equations is calculated in Equations (7) and (8), respectively;
(c) Proceed until the variation depicted in Figure 4 is consistent with the relationship between $t_{i}$ and $u_{i+1}$.

## 5. Numerical Simulation and Model Verification

### 5.1. Three-Point Bending Beam

**Figure 5:**
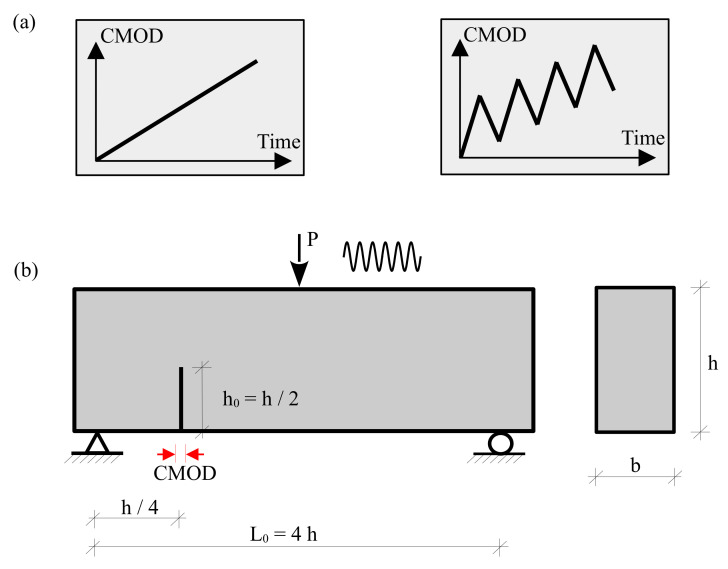
(**a**) Loading scenarios used in the simulation. Monotonic loading (**left**) and cyclic loading (**right**); (**b**) three-point bending beam for mixed mode crack propagation.

The mixed mode I-II in the TPB beam under monotonic and cyclic loading was predicted based on the numerical procedure presented in Figure 4. The experimental test results were done by Ref. [60] for concrete beams under mixed mode fracture. Table 1 summarizes the material parameters of the tested concrete specimens. The geometric dimensions of the specimen; TPB specimen with cross-section height of h=160 mm. The beam height was scaled to the beam length and span, as shown in Figure 5. The beam width is kept constant with b=80 mm. The notch depth was set to $h_{0}$ = h/2.

In the analyses, two systematic sets of loading scenarios are used. The first loading scenario introduces a typical monotonically increasing loading Figure 5a. In the second loading scenario, the sequence of unloading cycles are applied in Figure 5b.

**Figure 6:**
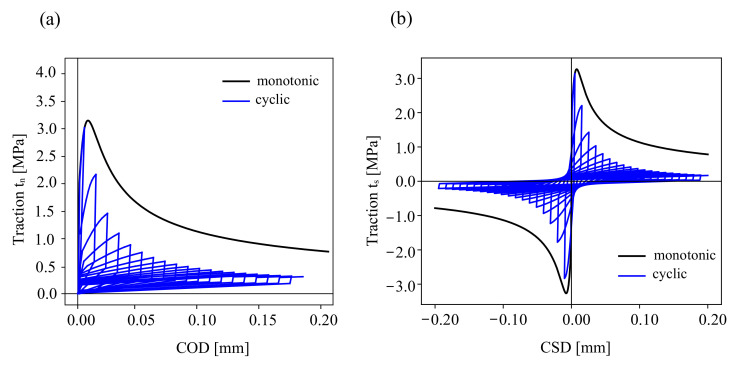
Cohesive traction response under cyclic loading (blue lines) and monotonic loading (gray lines) at the material point level: (**a**) traction-crack opening, (**b**) traction-crack sliding.

**Figure 7:**
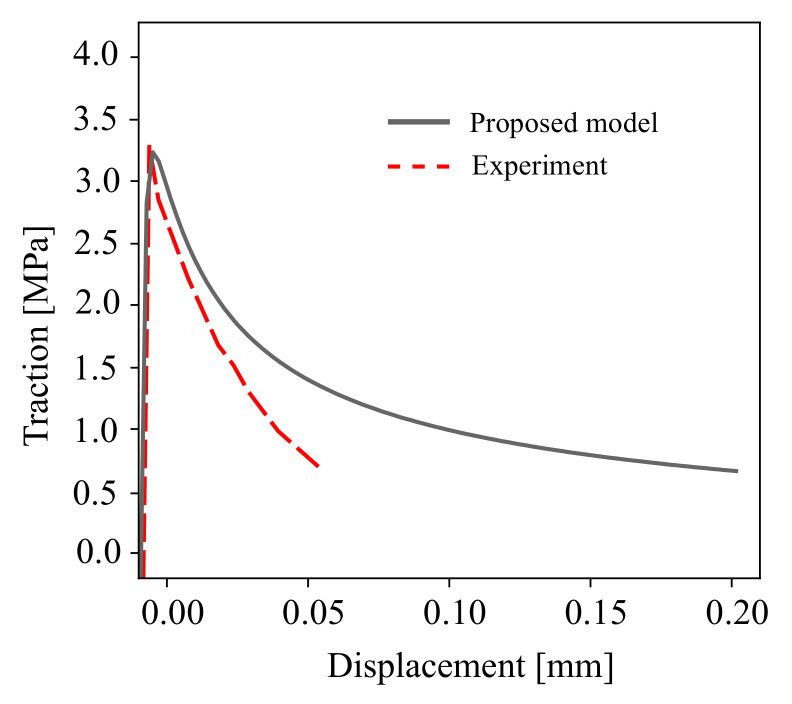
A comparison between the experimental measurements in Ref. [60] and the modelling results of the cohesive traction for mixed mode I-II fracture.

The properties of the concrete and cohesive interface element for COD and CSD responses are listed in Table 2. The proposed constitutive model has a set of plastic parameters γ,K and the damage strength *S*, as reported in Ref. [47]. In this study, the nonidentical material response of the proposed model and the experimental data is caused by the unified parametric prediction of the calibrated material behaviour for both monotonic and cyclic loading scenarios. In this calibration, the unified parameters of monotonic and cyclic material response at the Gauss point are plotted in Figure 6. A comparison of the experimental [60] and numerical simulations is depicted in Figure 7 for the traction stress curve of the mixed-mode crack displacement under monotonic loading. It should be noted that more advanced cohesive constitutive laws with coupled normal and tangential damage evolution can be used, e.g., see Ref. [61].

**Figure 8:**
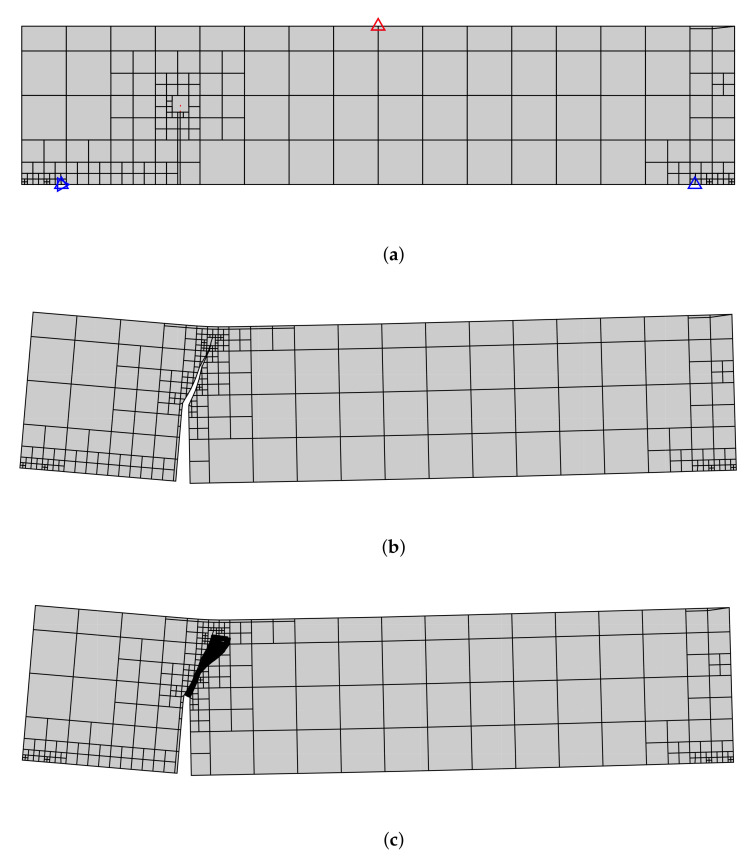
Predicted final crack paths of a TPB beam; (**a**) SBFEM mesh with 313 elements and boundary conditions, (**b**) predicted final crack path, (**c**) cohesive traction distribution.

The two-dimensional SBFEM modelling was utilized to establish the mesh of the TPB beam. A total of 205 elements were used. The mesh refinement near the crack tip was refined, as depicted in Figure 8a. Based on COD and CSD derived from the SBFEM calculation, the SIFs were computed. Eventually, the complete mixed mode I-II cyclic and monotonic crack propagation in TPB beam was simulated, as illustrated in Figure 8b. The crack propagation due to the increasing load is shown in Figure 8b. The results showed a curved crack path in the direction of the point of the external load (F). The distribution of the traction forces is shown in Figure 8c.

**Figure 9:**
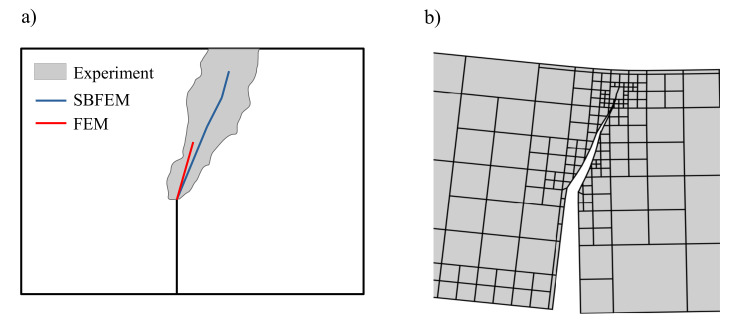
Predicted final crack paths of a TPB beam; (**a**) experimental shadow results in Ref. [60], (**b**) SBFEM simulation.

To demonstrate the effectiveness of the numerical method, the SBFEM results of test simulation were compared with the experimental results in Ref. [60]. Figure 9 shows the comparison of the crack propagation paths for monotonic loading, where the shaded region contains the experimentally measured crack paths. As can be shown, there is a reasonable agreement between the numerically predicted paths in SBFEM and both numerical FEM, as well as the experimental results in Ref. [60].

**Figure 10:**
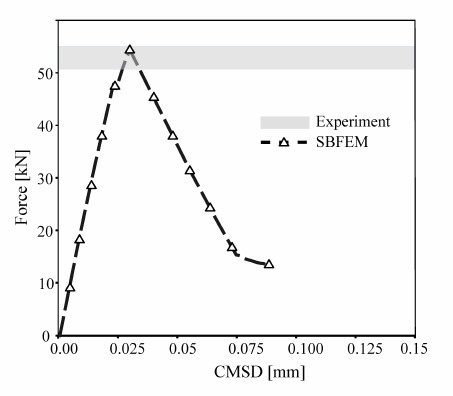
Numerical predictions of load-CMSD curves and the corresponding experimental data in Ref. [60] for the three-point bending test under monotonic loading.

Figure 10 compares the predicted load-crack mixed-displacement of the TBP beam with the experimental results reported by Ref. [60] under monotonic loading. The SBFEM numerical predictions’ related curve is shown in Figure 10, plotted in a black dashed line. The numerical results of the load-displacement curve are in good agreement with the experimental measurements. A maximum load of 53.1 kN is obtained at CMOD of 0.027 mm.

**Figure 11:**
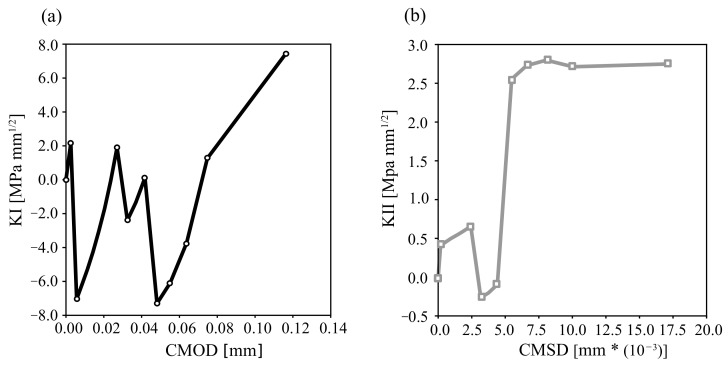
Mixedmode I–II crack displacement for monotonic loading: KI-CMOD (**a**) and KII-CMSD (**b**).

Figure 11 shows the results of the monotonic SIFs for both mode I and mode II, where the numerically measured SIFs are plotted. In Figure 11a, the points representing the initial mesh of Figure 10 are calculated once $K_{I}\geq 0$. Then, the crack opens gradually based on a crack propagation criterion. The numerical calculation of $K_{I}$ by SBFEM with a fewer number of degrees of freedom (DOFs) manifests good crack trajectory predictions.

**Figure 12:**
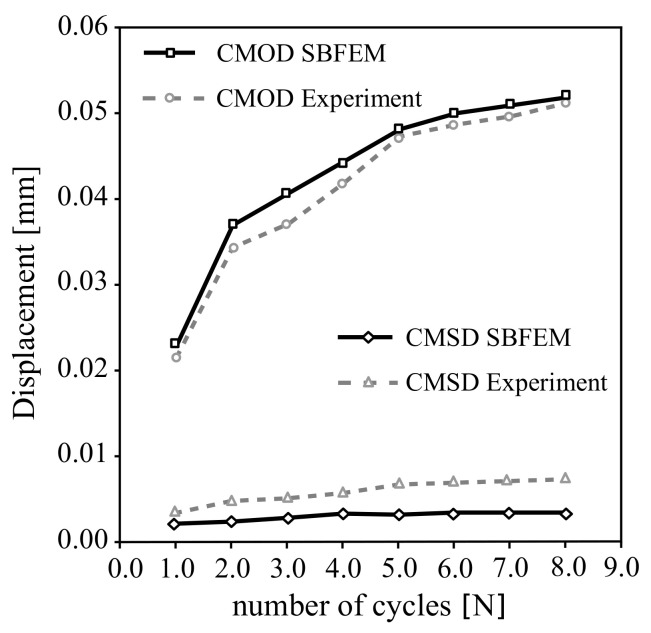
Crack displacement versus the number of cycles for experimental data in Ref. [60] and SBFEM; crack mouth opening CMOD and sliding CMSD.

The experimental observations and numerical calculations for a cyclically mixed-mode loading are shown in Figure 12. The loading is controlled by the CMOD/CMSD, including eight unloading cycles. The results show that the numerical comparison of crack propagation CMSD is relatively stable while the CMOD increases as the N of the cycle increases. As a result, there is a good agreement between the experiment results and the numerical predictions of CMSD in Figure 12.

### 5.2. Four-Point Bending Beam

**Figure 13:**
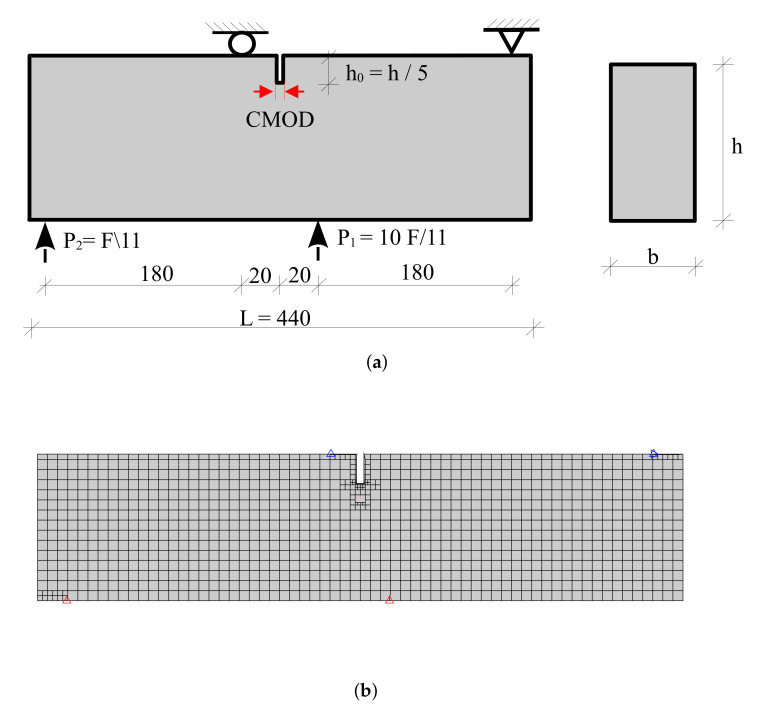
A single-notched concrete beam under monotonic mixed-mode loading. (**a**) Geometry, (**b**) initial mesh with boundary conditions.

The proposed method is next verified using the numerical results of the four-point bending concrete beam under mixed mode fracture [62]. The geometry, loads, and support conditions are illustrated in Figure 13. The width and height of the specimen are denoted by 100 × 100 [mm]. The material properties of the concrete beam are summarized in Table 3.

A particular loading condition is implemented to generate mode II crack initiation for both monotonic and cyclic loading. The calculation is performed under displacement-controlled loading. For the analysis of the concrete beam, the following material properties are used, see Table 4 below.

**Figure 14:**
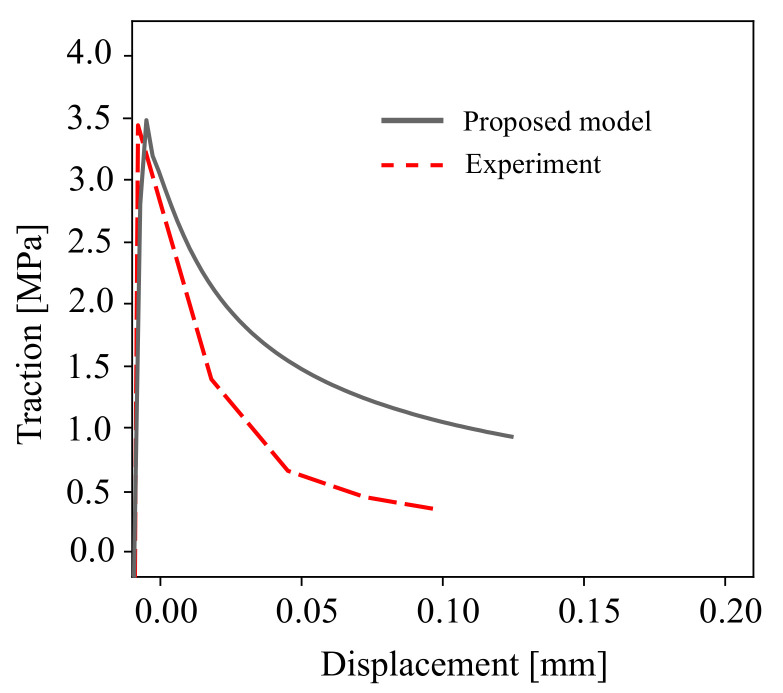
Comparison of the tested beam in Ref. [62] and the proposed cohesive traction for mixed modes I–II fracture.

The initial mesh of the tested beam is illustrated in Figure 13. The mesh consists of 1069 elements. The crack propagation length of Δa=22 mm is adopted in the crack propagation simulation. Figure 14 compares the predicted traction point displacement response of the developed method with the numerical results of Ref. [62] in the literature. Overall, there is good agreement between the results of the developed method and the experimental measurements. The pre-peak response compares very well with the previously reported numerical results. The numerical SBFEM predicted a maximum load of 36.2 kN and is closest to both the experimental and FEM predictions of Ref. [62].

**Figure 15:**
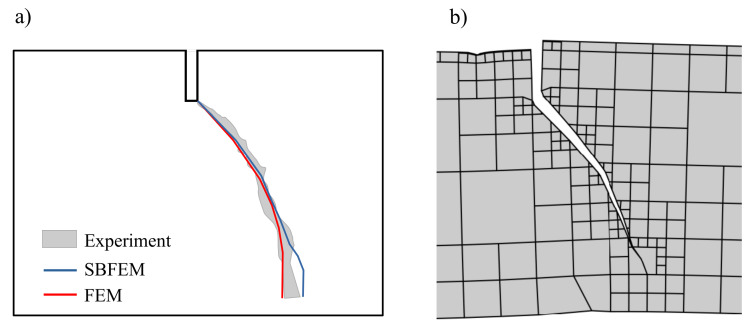
Predicted final crack paths of a tested beam; (**a**) the experimental shadow results in Ref. [62] and the numerical results and (**b**) SBFEM simulation.

Figure 15 represents the predicted crack propagation process. During the simulation, a crack propagates from the tip notch towards the loading point on the bottom surface of the four point bending beam. The paths of the crack in the experimental results and SBFEM are curved. In Figure 15, the predicted crack path of the FEM simulation in Ref. [62] seems to be more efficient, however, the mesh adaptive procedure of the SBFEM will produce more efficient results and reduce the computational time costs.

**Figure 16:**
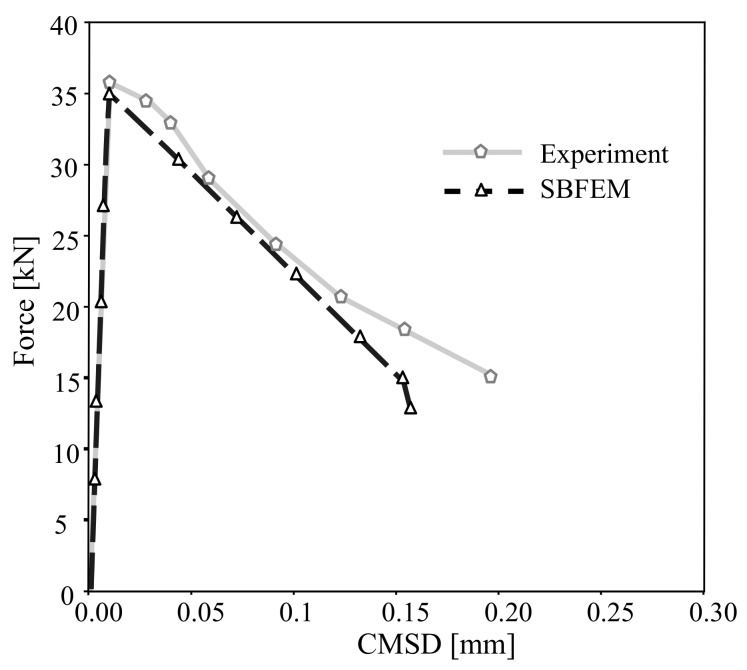
Numerical predictions of load-CMSD curves and the corresponding experimental data in Ref. [62] for the tested beam under monotonic loading.

Figure 16 compares the predicted load-crack displacement response of the developed method with the numerical FEM results of Ref. [62]. Overall, the results obtained from the developed method agree well with the numerical for the monotonic loading. The pre-peak response compares well with the previously published numerical results. All the numerical predictions below estimate the maximum load. The SBFEM predicted a maximum load of 34.8 kN and is closest to the FEM predictions.

**Figure 17:**
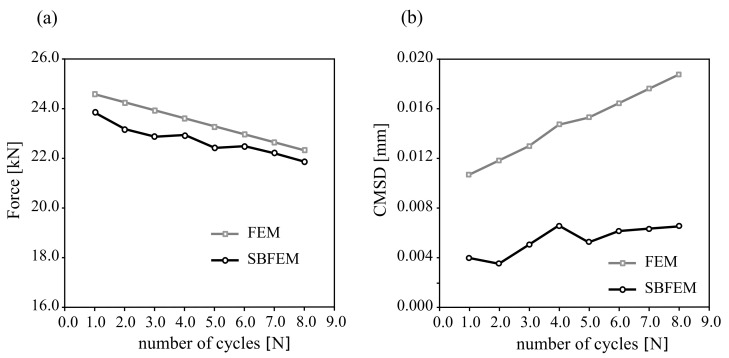
SBFEM numerical predictions of (**a**) load-CMSD curves and (**b**) crack displacement of the corresponding finite element data for tested beam under cyclic loading in Ref. [62].

Figure 17 shows SBFEM numerical predictions and FEM results for a cyclically mixed-mode loading. The loading is controlled by the CMSD, also including eight unloading cycles applied until failure. The results in Figure 17a show that the traction forces of SBFEM have a very good agreement with the numerical results in FEM. In Figure 17b, the crack displacement of the SBFEM simulation is more underestimated in comparison to the FEM measurements. The calculation of the mixed crack displacement at the material point, the number of load steps, and the democratization of the applied mesh have a significant effect on the SBFEM numerical results. However, more experimental data of cyclic mixed mode tests are required to validate the proposed numerical method.

## 6. Conclusions

In this paper, a newly developed SBFEM numerical method for mixed mode crack propagation in concrete under cyclic loading was proposed. The proposed procedure allowed accurate SIFs to be calculated directly from the SBFEM analytical framework without more discretization at crack-tip meshes or by using singular elements, as in FEM. Comparing the thermodynamic softening law of the constitutive model for fracture, several aspects have been provided, which incorporate the loading-unloading path, the damage evolution during the load cycle, and the crack traction displacement behaviour.

The cyclic behaviour of interfaces using SBFEM has been successfully described using the damage accumulation hypothesis. The proposed method showed the ability to simulate both monotonic and cyclic behaviour of a cohesive crack interface element, e.g., concrete interface, utilizing a consistent set of material parameters. The cyclic loading simulations’ output agreed well with experimental data from the literature. The proposed method performed to study the effect of fatigue loading provides promising results and establishes a damage accumulation hypothesis for the simulation of multiple cohesive cracks under 1000 load cycles.

## Figures and Tables

**Table 1:** The parameters of the material of the experimental test in Ref. [60].

Parameter	Denomination	Value	Unit
$f_{c}$	Compressive strength	44.24	[MPa]
$f_{ct}$	Tensile strength	3.35	[MPa]
$E_{c}$	Young’s Modulus	35.38	[GPa]
ν	Poisson ratio	0.21	[-]

**Table 2:** Model parameters for the concrete cohesive interface element.

Parameter	Denomination	Value	Unit
*E*	Elastic cohesive modulus	3000	[MPa]
$\bar{\sigma }$	Reversibility limit	2.0	[MPa]
*K*	Isotropic hardening modulus	400.0	[MPa]
γ	Kinematic hardening modulus	500.0	[MPa]
*S*	Damage strength	0.25 × $10^{-4}$	[MPa]
*r*	Damage accumulation parameter	1.0	[-]
*c*	Damage accumulation parameter	2.0	[-]

**Table 3:** Parameters of the material in the experimental test by Ref. [63].

Parameter	Denomination	Value	Unit
$f_{ct}$	Tensile strength	3.44	[MPa]
$G_{f}$	fracture energy	0.126	[N/mm]
$E_{c}$	Young’s Modulus	30.0	[GPa]
ν	Poisson ratio	0.20	[-]

**Table 4:** Model parameters for the concrete cohesive interface element.

Parameter	Denomination	Value	Unit
*E*	Elastic cohesive modulus	3500	[MPa]
$\bar{\sigma }$	Reversibility limit	2.0	[MPa]
*K*	Isotropic hardening modulus	400.0	[MPa]
γ	Kinematic hardening modulus	500.0	[MPa]
*S*	Damage strength	0.25 × $10^{-4}$	[MPa]
*r*	Damage accumulation parameter	1.0	[-]
*c*	Damage accumulation parameter	2.0	[-]

## Data Availability

Not applicable.

## Nomenclature

${\dot{u}}^{P}$	Relative displacement	ω	Damage variable
w=COD	Crack opening at material point	*Y*	Energy release rate
s=CSD	Crack sliding at material point	*S*	Damage strength parameter
c,r	Exponential damage parameters	$\sigma_{n},\bar{\sigma }$	Effective stress limit
*m*	Material constant	*E*	Elastic stiffness
α	Hardening material variable	*E*	Elastic stiffness
$\sigma =t_{n}$	Cohesive normal stress	$\tau =t_{s}$	Cohesive tangential stress
$E^{lag}$	Element stiffness of the interface	γ,K	Isotropic and kinematic hardening moduli
η,ξ	Local coordinate system of SBFEM	$\bar{\lambda_{i}}$	Eigenvalue matrices
{u}	Displacement field	*D*	Material constitutive matrix
N(η)	Nodal shape function	$K_{pol}$	Stiffens matrix of the domain
$\bar{\phi_{i}}$	Eigenvector matrices	$c_{i}$	Integration constants of the SBFEM
$\left[B_{1}\right],\left[B_{2}\right]$	Strain-displacement matrices of SBFEM system	*M*	Number of displacement modes
*P*	External applied force	*N*	Number of load cycles
θ	Crack propagation angle	$\Delta_{a}$	Crack propagation length
$L_{0}$	Crack length	$K_{int}$	Stiffens matrix of interface element
*A*	Crack surface area	$w_{i}$	Gaussian weight function
CMSD	Crack mouth sliding displacement	$F_{t}$	Nodal side face load
CMOD	Crack mouth opening displacement	$K_{I},K_{II}$	Crack mode I & mode II stress intensity factors
{σ}	Stress field
